# Can Pillow Use Prevent Postdural Puncture Headache? A Critical Review

**DOI:** 10.1155/anrp/3820438

**Published:** 2026-10-14

**Authors:** Aragaw Hamza Yimer, Ahmed Muhye Seid

**Affiliations:** ^1^ Department of Anesthesia College of Medicine and Health Sciences Dire Dawa University Dire Dawa Ethiopia ddu.edu.et; ^2^ Department of Public Health College of Medicine and Health Sciences Dire Dawa University Dire Dawa Ethiopia ddu.edu.et

## Abstract

Postdural puncture headache (PDPH) is a recognized complication of neuraxial anesthesia that follows unintentional dural puncture during epidural techniques or intentional dural puncture during spinal anesthesia and other neuraxial procedures. It has traditionally been managed with a period of mandatory supine bed rest and pillow restriction, a practice grounded in the historical belief that recumbency limits ongoing cerebrospinal fluid (CSF) leakage, thereby preventing headaches. This “no‐pillow” instruction remains widely taught and practiced, including among clinicians, despite an absence of literature support and evidence suggesting the opposite may be true. This review synthesizes the current literature, including randomized controlled trials, Cochrane systematic reviews, and multisociety clinical guidance, to critically examine whether pillow restriction after dural puncture reduces PDPH incidence. Evidence indicates that neither prolonged bed rest nor pillow avoidance reduces PDPH incidence and that enforced postural restriction instead increases patient discomfort, back and neck pain, and the risk of complications associated with prolonged immobilization. This review advocates the revision of institutional protocols to permit pillow use and early postoperative mobilization, as tolerated.

## 1. Introduction

According to the third edition of the International Classification of Headache Disorders (ICHD‐3), published by the International Headache Society in 2018, postdural puncture headache (PDPH) is defined as a headache attributed to low cerebrospinal fluid (CSF) pressure, occurring within five days of a lumbar puncture and caused by CSF leakage through the dural puncture site [1]. PDPH is a recognized complication following unintentional dural puncture during epidural analgesia or intentional dural puncture performed for spinal anesthesia and other diagnostic or interventional neuraxial procedures. Its reported incidence varies widely, from < 2% to as high as 40%, depending on the procedural technique and patient‐specific factors [2–4].

This variability is substantially driven by the technique used to access the subarachnoid or epidural spaces. Inadvertent dural puncture during epidural anesthesia is a more frequent cause of PDPH than deliberate dural puncture during spinal anesthesia because spinal anesthesia typically employs small pencil‐point needles designed to minimize dural trauma. During epidural catheter placement, inadvertent puncture of the dura occurs in approximately 1.5% of cases, and more than half of these patients (52.1%) subsequently develop PDPH [5]. More recent data suggest that the incidence of PDPH after dural puncture with a larger epidural needle may be as high as 76%–85% [6].

PDPH is typically postural in nature, presenting within the first 5 days after a witnessed or suspected dural puncture [7]. The headache is classically fronto‐occipital, radiating to the neck or shoulder region, and characteristically worsens within minutes of assuming an upright posture while improving on lying flat; symptoms may also be exacerbated by coughing or a Valsalva maneuver [2, 8]. The headache is frequently accompanied by neck stiffness, subjective hearing disturbance, visual disturbance, and vertigo [9]. Although PDPH often resolves spontaneously within 2 weeks, its severity can interfere with activities of daily living, a particular concern for postpartum patients who must care for a newborn, and it has been associated with more serious complications, including subdural hematoma, cerebral venous sinus thrombosis, cranial nerve dysfunction, depression, and chronic headache or backache [8].

The primary risk factors for PDPH include patient age, sex, body mass index, and prior headache history [10]. Independent risk factors identified are female sex, younger age, pregnancy, vaginal delivery, low body mass index, and nonsmoking status [2]. While headaches are common in the postpartum period, only a minority are due to PDPH, necessitating careful clinical evaluation for accurate diagnosis [11].

In 2021, the American Society of Anesthesiologists (ASA) stated that the diagnosis of PDPH is based on clinical presentation, thorough history, and physical examination, focusing on symptoms such as neck stiffness, spinal pain, auditory issues, visual disturbances, and vertigo [12].

Despite this well‐characterized clinical picture, the standard preventive strategy taught in many training programs and practiced in many institutions including widely among clinicians remains mandatory supine bed rest with strict avoidance of a pillow, based on the premise that this positioning reduces hydrostatic CSF pressure at the puncture site and thereby limits ongoing leakage [13, 14]. This practice persists despite a substantial body of modern evidence, including randomized controlled trials and multisociety clinical guidelines, indicating that such positioning does not reduce PDPH incidence and may instead increase patient discomfort, back pain, and other complications associated with prolonged immobilization [13, 15]. This review synthesizes the current literature to critically evaluate the “no‐pillow” dogma and to advocate for early postoperative mobilization, guided by patient comfort, as an alternative approach.

## 2. Pathophysiology

In 1899, August Bier performed one of the earliest documented spinal anesthetics on himself and was among the first to describe PDPH, proposing that it resulted from loss of CSF [16]. Although the precise mechanism remains incompletely understood, the leading explanation holds that PDPH results from reduced CSF pressure caused by ongoing loss of fluid into the epidural space through the dural puncture site [15]. CSF leakage that exceeds the rate of CSF production causes CSF pressure to fall, producing a state of CSF hypotension, and it is estimated that loss of more than 10% of the total CSF volume is sufficient to produce an orthostatic headache [15].

Two complementary mechanisms have been proposed to explain how CSF hypotension causes headache. The first, the “sagging” theory, holds that in the upright position the reduced volume of CSF is pulled caudally from the cranial cavity into the vertebral canal, causing the brain to sag toward the foramen magnum and placing traction on the meninges and cranial nerves; this theory accounts for the cranial nerve palsies occasionally observed in patients with PDPH [15]. The second proposed mechanism is compensatory cerebral vasodilation, occurring in response to CSF loss in order to maintain a constant total intracranial volume, which is thought to produce a headache with a vascular character [15].

In a minority of patients with PDPH, signs of intracranial hypotension are evident in brain imaging. The characteristic manifestations of reduced CSF volume can be summarized as mnemonic SEEPS: sagging of the brainstem, enhancement of the dura, engorgement of venous structures (particularly distension of the transverse venous sinus), pituitary hyperemia, and subdural fluid collections [17]. Subdural hygromas or hematomas may develop following dural puncture, as a passive accumulation of fluid in the subdural space occurs in accordance with the Monro–Kellie doctrine as intracranial CSF volume falls [18].

## 3. The “No‐Pillow” Dogma: Historical Rationale

The historical justification for mandatory supine positioning without a pillow is primarily based on hydrostatic principles, which are believed to reduce hydrostatic pressure at the dural puncture site, alleviating the symptoms of PDPH, which worsen when upright. Consequently, bed rest and supine positioning have been traditionally advocated to relieve symptoms and prevent PDPH, although any symptom relief is usually temporary and no proven benefit for prevention exists [19, 20].

Omitting a pillow is commonly believed to reduce pressure at the puncture site by keeping the head level with the spine, but this assumption lacks physiological support [13, 15]. Evidence shows that head elevation from a standard pillow has a negligible effect on intracranial pressure compared with a fully upright position, and there is no literature on the practice of avoiding pillows. In fact, the evidence suggests otherwise [13].

## 4. Bed Rest, Posture, and PDPH Incidence

The bed rest and supine positions have traditionally been used to treat and prevent PDPH symptoms. However, evidence from randomized controlled trials and systematic reviews indicates that bed rest has little to no effect on preventing or reducing PDPH severity and should not be recommended for this purpose [19, 20].

A 2013 and 2016 Cochrane systematic review found no evidence that bed rest prevents PDPH and suggested that it might even increase the risk compared to early mobilization [19, 21]. Additionally, a commentary in the literature supported these findings, stating that prolonged bed rest does not decrease headache incidence [22].

Park et al. conducted a systematic review and meta‐analysis of spinal anesthesia, revealing no significant difference in the incidence of PDPH between patients with 24‐h bed rest and those who ambulated early [23]. Similarly, a randomized controlled trial by Thornberry and Thomas found no protective benefit from strict positioning after dural puncture in obstetric patients [20]. This evidence highlights a disconnect between the physiological assumptions behind the no‐pillow practice and actual outcomes observed in trials.

A randomized trial by Maharjan assessed the effects of pillow use after spinal anesthesia in 138 patients. Patients were split into two groups: one using a pillow and one without, for 8 h postsurgery. No significant differences were observed in the incidence of postural headache (PDPH), with no cases observed in either group. However, the pillow group reported enhanced comfort and fewer complaints of neck stiffness and back pain [24].

Shahin et al. found that pillow use among cesarean section patients is linked to a slight increase in ordinary, nonpostural headaches but does not significantly affect the incidence of true PDPH [25]. This distinction is clinically relevant because ordinary headaches are managed differently than true PDPH, which is associated specifically with CSF leakage [24, 25]. These findings challenge the widespread clinical practice of lying flat without a pillow to protect against PDPH. The literature does not support this practice, and controlled evidence indicates that pillow restriction increases discomfort without reducing the risk [13, 24].

## 5. Intraprocedural and Postprocedural Bed Rest

An important distinction that is frequently overlooked in clinical teaching is the difference between a patient’s position during the dural puncture itself and its position afterward [26]. These are physiologically distinct questions, and evidence supporting one should not be used to justify practice regarding the other.

A randomized controlled trial by Sharma et al. revealed that the lateral decubitus position during spinal anesthesia for lower‐segment caesarean sections significantly reduced the incidence of PDPH compared to the sitting position [26]. This indicates that the intraprocedural position may influence the trajectory of the needle in relation to dural fibers, thereby affecting the risk of PDPH. Conversely, literature on bed rest suggests that posture and duration after dural puncture do not significantly affect the occurrence of headache [19, 20].

Recent literature indicates that extended bed rest or rigid postprocedural positioning is unnecessary for preventing PDPH, with evidence suggesting that shorter bed rest might be more beneficial [19, 23]. It warns against confusing intraprocedural positioning with postprocedural guidelines, which lack support, emphasizing the need to re‐evaluate current institutional practices regarding this issue [19, 26].

## 6. Adverse Effects of Prolonged Postural Restriction

In addition to the absence of any demonstrated preventive benefits, enforced supine positioning without a pillow carries the recognized costs of its own. Restricting a patient’s posture and movement creates problems distinct from PDPH itself, including headache and pain localized to the head, cervical spine, shoulders, and back; increased risk of respiratory and urinary tract infection; increased risk of deep vein thrombosis; pressure ulcers; and difficulty with feeding, urination, defecation, personal hygiene, and even conversation [27–30].

A randomized controlled trial indicated that patients who were immobilized after spinal anesthesia experienced a significantly higher incidence of neck, back, and shoulder pain than those who were mobilized early. Additionally, prolonged immobilization is linked to complications such as delayed bowel and bladder function, increased nursing workload, longer hospital stays, and lower patient satisfaction, without any proven benefit in reducing PDPH risk [29, 30].

Multisociety clinical guidelines increasingly recommend moving away from postural restriction after dural puncture and emphasize early mobilization instead [12, 13]. Current guidance holds that bed rest should not be routinely prescribed as a preventive strategy against PDPH. While lying supine may offer transient symptomatic relief once a postural headache has already developed, it does not prevent the onset of PDPH in the first place, and this distinction between treatment and prevention is frequently blurred in traditional teaching [13, 19]. Immediate mobilization, as tolerated, is instead encouraged to help patients return to normal activity and to avoid the discomfort and complications associated with prolonged bed rest [23].

Providing patients with a pillow for head support after spinal anesthesia enhances comfort and does not increase the risk of PDPH. This recommendation contradicts traditional advice against pillow use, suggesting that hospitals and anesthesiologists should adopt evidence‐based, patient‐centered care protocols [13, 19].

## 7. Limitations of the Current Evidence

Several limitations of the current evidence base are noted, including small and methodologically varied studies involving obstetric populations. The key trial specifically addressing pillow use was not adequately powered to detect differences in PDPH incidence, although it was adequately powered to detect differences in comfort.

To enhance the evidence for pillow‐use recommendations, larger randomized trials are necessary, utilizing contemporary small‐gauge needles across both obstetric and nonobstetric surgical populations [19, 23, 24].

## 8. Conclusion

The use of routine supine bed rest without a pillow after dural puncture lacks randomized evidence supporting its effectiveness in preventing PDPH. The rationale behind this practice is challenged by controlled evidence, which indicates that head elevation does not significantly affect CSF leakage. Allowing a pillow for head support after spinal anesthesia is reasonable, enhances comfort, and minimizes musculoskeletal issues from extended immobility. Clinicians should differentiate between intraprocedural and postprocedural positioning, as the former has some evidence to support it, unlike the latter. These insights advocate a shift to patient‐centered care and reassessment of established protocols, including the no‐pillow guideline, in response to new evidence.

## Funding

No funding was received for this manuscript.

## Conflicts of Interest

The authors declare no conflicts of interest.

## Data Availability

The data that support the findings of this study are available on request from the corresponding author. The data are not publicly available due to privacy or ethical restrictions.
